# Diagnostic Yield of Ultrasound-Guided Omental Biopsy for Omental Thickening Using Histopathology As the Reference Standard

**DOI:** 10.7759/cureus.101569

**Published:** 2026-01-14

**Authors:** Quratulain Haroon, Misbah Tahir, Shaiq Hussain, Danial Khalid, Khalid Mustafa, Jawaid Iqbal

**Affiliations:** 1 Radiology, Liaquat National Hospital and Medical College, Karachi, PAK; 2 Interventional Radiology, Liaquat National Hospital and Medical College, Karachi, PAK; 3 Radiology, Ziauddin University, Karachi, PAK

**Keywords:** ct scan abdomen, diseases patterns, omental biopsy, omental caking, peritoneal carcinomatosis, peritonitis, solitary peritoneal thickening, ultrasound

## Abstract

Background

Histopathological evaluation is vital in distinguishing between benign and malignant lesions. Assessment of the different patterns of peritoneal lesions on CT scans may help to differentiate inflammation and malignancy. It utilizes various imaging modalities, among them, ultrasound-guided percutaneous biopsies are minimally invasive, have low complications, and are cost-effective.

Objective

To assess different disease patterns by ultrasound-guided omental biopsy in patients with solitary peritoneal thickening found on CT scans, using histopathology as the reference standard.

Study design, setting, and duration

This was a prospective, cross-sectional study, carried out for over 2.5 years in the Department of Radiology at Liaquat National Hospital, Karachi, Pakistan.

Material and methods

The study included a total of 167 patients. Biopsies were conclusive in 144 patients, so they were included in the study. In 23 patients, biopsies were non-conclusive, so they were excluded. The thickness, echo-texture configuration, and presence of nodules within the thickened larger omentum were all investigated and appraised under ultrasound supervision. A local anesthetic was infiltrated into the abdominal wall. The biopsy needle path was carefully assessed with the color Doppler ultrasound to avoid blood vessels. Biopsy samples were taken, and considered successful if the definitive diagnosis of whether benign or malignant was made on histopathology.

Results

Of the total patients, 54 (37.5%) were men and 90 (62.5%) were women. The mean age was 48.69±16.17 years. Disease pattern showed 93 (64.6%) peritoneal carcinomatosis, 26 (18.1%) tuberculous peritonitis, 15 (10.4%) chronic inflammation, seven (4.9%) mesothelial hyperplasia, and five (3.5%) panniculitis. There was a highly significant association between age group and peritoneal carcinomatosis (p<0.001) and tuberculous peritonitis (p<0.001). Similarly, chronic inflammation was significantly associated with the duration of symptoms (p=0.014).

Conclusion

Ultrasound-guided omental biopsy is a safe, minimally invasive, and highly effective diagnostic tool for evaluating omental thickening. It provides a high diagnostic yield with minimal complications, often precluding the need for more invasive surgical procedures like diagnostic laparoscopy. Our findings support its use as a primary diagnostic modality for obtaining a definitive histopathological diagnosis.

## Introduction

The greatest peritoneal fold, the greater omentum, is imperceptible on imaging in its normal form and only noticeable when illnesses like inflammation and cancer are present [1]. Omental abnormality can be readily detected with CT and/or ultrasound (US) imaging [2]. Assessment of the different patterns of peritoneal lesions on CT scans may help to differentiate inflammation and malignancy. Smooth peritoneal thickening is most commonly associated with inflammation, and the nodular pattern is mostly seen in malignancies. However, imaging features can overlap, making it challenging to differentiate between them [1,2].

Making an accurate diagnosis and selecting the most effective treatment plan depends on exploring the cause of peritoneal thickness. Image-guided biopsy has gained popularity because of its minimally invasive nature, low complications, and cost-effectiveness [3,4]. In order to distinguish between benign and malignant tumors, and to prevent the morbidity that comes with laparoscopic surgery for tissue diagnostics, US-guided percutaneous biopsy is widely used [5,6].

Because of their location in the anterior peritoneum, omental abnormalities are easily detectable by ultrasonography or CT imaging and are easily reachable for percutaneous biopsy [7]. However, a number of variables, such as the depth of the lesions, their proximity to veins, the gastrointestinal system, or solid organs, and their comparative mobility in comparison to lesions within solid organs, might occasionally make the surgery technically challenging [8,9].

Positron Emission Tomography/Computed tomography (PET/CT) or fluorine-18-fluorodeoxyglucose (18F-FDG) PET is a useful imaging technique that aids in the differentiation of peritoneal pathology. Although it is not totally reliable, a higher standardized uptake value (SUV) of more than 5.1 may aid in distinguishing benign peritoneal inflammation from peritoneal carcinomatosis [10,11].

A comparatively high diagnosis rate has been shown for laparoscopic peritoneal biopsy [9]. However, laparoscopic procedures involve invasive manipulation, which may increase the risk of complications, thereby necessitating the administration of anesthesia in a controlled operating room setting [11,12]. In contrast, peritoneal lesion biopsies performed under US guidance can be quicker, safer, and less invasive.

This study aimed to evaluate the diagnostic value and safety of US-guided omental biopsy for assessing various disease patterns in cases of solitary peritoneal thickening visible on CT scans. US-guided omental biopsy facilitates early disease diagnosis and management, thereby avoiding unnecessary surgeries. Notably, there is a paucity of international studies investigating the biopsy of peritoneal thickening of intermediate cause, particularly when visualized as fat infiltration on abdominal scans.

## Materials and methods

Study design and data collection

Eligible participants who were referred to the interventional radiology department of Liaquat National Hospital and Medical College, Karachi, Pakistan, to undergo US-guided omental biopsy for suspected peritoneal disease were enrolled in the study. The inclusion criteria were age >16 years, presence of ascites or peritoneal thickening on imaging studies, and clinical suspicion of peritoneal carcinomatosis, tuberculous peritonitis, or other peritoneal diseases. Exclusion criteria included patients with bleeding diathesis or coagulopathy, pregnancy or lactation, or a previous history of abdominal surgery or peritoneal dialysis.

The study was conducted after obtaining necessary authorization from the Ethical Review Committee of the Liaquat National Hospital and Medical College, ensuring adherence to ethical standards (approval no. 0604-2021-LNH-ERC). Additionally, informed consent was obtained from each patient prior to participation in the study.

**Figure 1:**
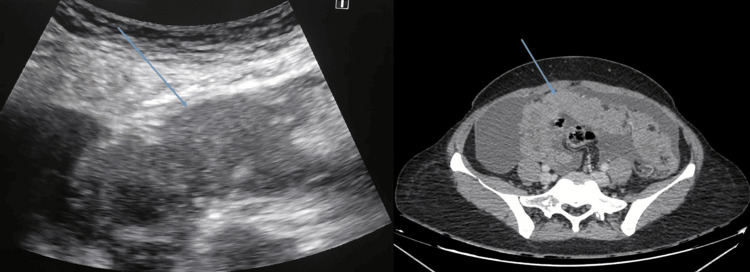
Ultrasound image and CT scan of one of the patients Ultrasound image showing omental thickening (blue arrow). CT scan abdomen of the same patient showing omental thickening and ascites (blue arrow).

Platelets, prothrombin time (PT), and international normalized ratio (INR) were measured as part of the blood coagulation profile. Prior to biopsy, the location of the thickest peritoneal lesion on the abdominal CT scan was determined, and its thickness, configuration, echo texture, and presence of omental nodules were assessed using greyscale ultrasonography (Figure 1).

**Figure 2:**
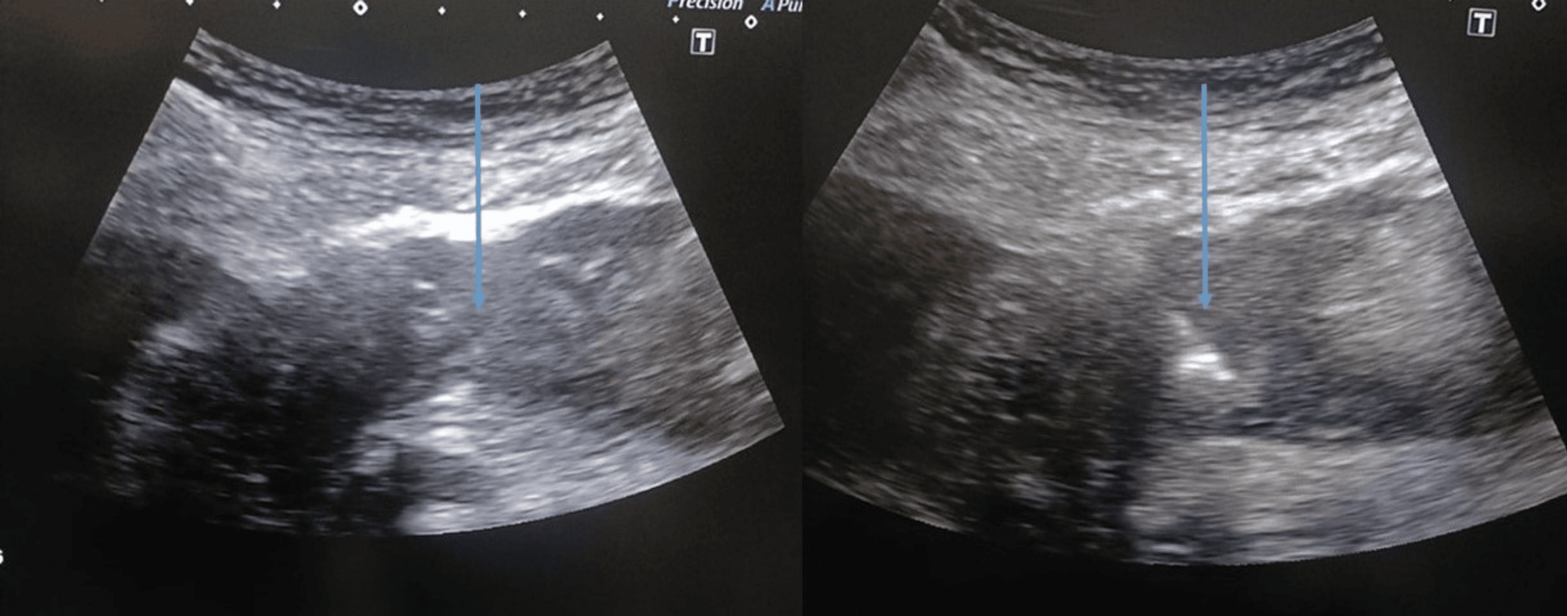
Ultrasound guided needle placement within the thickened omentum (needle tip indicated by blue arrow)

To differentiate between the intestinal wall and larger omentum, the probe was applied to the abdominal wall without applying pressure. The thickest part (>1 cm) of the greater omentum, which is close to the abdominal wall, was selected and designated as the intended needle entrance site, particularly the area where nodules or masses were discovered. Povidone iodine solution was used to clean the biopsy's intended needle entrance site and the surrounding area. Using a 21-gauge needle, a local anesthetic lignocaine HCL 2% solution 10ml, was injected subcutaneously into the abdominal wall. All core biopsies were carried out with the coaxial approach, 18-gauge Tsunami needles (Tsunami Medical, Modena, Italy) passed through a 16-gauge lumbar puncture needle (Becton, Dickinson and Company, Franklin Lakes, NJ, USA). The Aplio 300 (Canon Medical Systems, Otawara, Japan) with a 3.5MHz convex or a 12MHz linear array transducer served as the guiding US system. The researcher and one doctor with more than five years of radiology experience performed the biopsy. To avoid blood vessels, the needle path was evaluated using the color Doppler US (Figure 2).

Following each procedure, the patient was monitored for four hours in the recovery area to check vital signs and look for any potential problems. A senior histopathologist reported the results of the histopathology, which was completed in the histopathology lab. If the pathologist made a specific benign or malignant diagnosis, the biopsy was deemed successful. A diagnosis was made and noted on a proforma following the receipt of the results. A premade proforma was used to collect all data.

Data analysis

Data analysis was conducted using IBM SPSS Statistics for Windows, Version 25 (Released 2017; IBM Corp., Armonk, New York, United States). The variables that were based on classification were expressed as frequencies and percentages. For categorical variables, the mean ± SD was computed. To observe the impact of effect modifiers on outcome variables, age, symptom duration, and gender stratification were used. The post-stratification chi square test was used, with a p-value of less than 0.05 being considered significant.

## Results

In our study, 144 patients aged between 16 and 80 years, of either gender, meeting the study's inclusion criteria, were included to assess the different patterns of disease by US-guided omental biopsy in patients with solitary peritoneal thickening found on CT scan. There were 54 male patients (37.5%) and 90 female patients (62.5%), as presented in Table 1.

**Table 1:** Demographic characteristics of the patient population (n=144)

Variables	n (%)
Gender	
Male	54 (37.5)
Female	90 (62.5)
Age (years)	
Mean ± Standard deviation (SD)	48.69±16.17
Age groups	
≤45 years	56 (38.9)
>45 years	88 (61.1)
Duration of symptoms (days)	
Mean ± SD	30.85±52.84
Duration of symptoms	
≤30 days	29 (20.1)
>30 days	115 (79.9)

The overall mean age was 48.69±16.17 years. The age was further stratified into two groups, as presented in Table 1. The mean duration of symptoms was 30.85±52.84 days. Table 1 shows the two groups into which the duration of symptoms was further split.

The disease pattern on the US-guided peritoneal biopsy was 64.6% peritoneal carcinomatosis, 18.1% tuberculous peritonitis, 10.4% chronic inflammation, 4.9% mesothelial hyperplasia, and 3.5% panniculitis. The frequency distribution of the disease pattern is presented in Figure 1.

**Figure 3:**
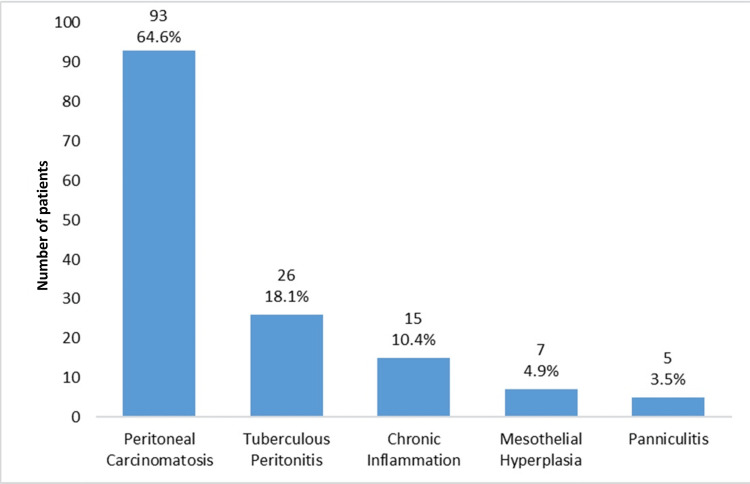
Pattern of diseases by ultrasound guided biopsy in patients with solitary peritoneal thickening on CT scan

Using gender, age group, and illness duration as parameters, stratification was carried out. Gender showed statistically significant association with peritoneal carcinomatosis (p=0.001), tuberculous peritonitis (p=0.019), and chronic inflammation (p=0.014). The age group showed highly significant correlation with peritoneal carcinomatosis (p<0.001) and tuberculous peritonitis (p<0.001). The association between duration of symptoms and chronic inflammation was also statistically significant (p=0.014). The detailed association of disease pattern with the age group, gender, and duration of symptoms is presented in Tables 2, 3.

**Table 2:** Association of peritoneal carcinomatosis, tuberculous peritonitis, and chronic inflammation with the demographic profile of the patients †Fisher exact test was applied; *Significance at 0.05 level

Variables	Group	Peritoneal carcinomatosis			Tuberculous peritonitis			Chronic inflammation		
		Yes	No	p-value	Yes	No	p-value	Yes	No	p-value
		n (%)	n (%)		n (%)	n (%)		n (%)	n (%)	
Gender	Male	26 (28)	28 (54.9)	0.001*	15 (57.7)	39 (33.1)	0.019*	10 (66.7)	44 (34.1)	0.014*
	Female	67 (72)	23 (45.1)		11 (42.3)	79 (66.9)		5 (33.3)	85 (65.9)	
Age group	≤45 years	25 (26.9)	31 (60.8)	<0.001*	19 (73.1)	37 (31.4)	<0.001*	8 (53.3)	48 (37.2)	0.225
	>45 years	68 (73.1)	20 (39.2)		7 (26.9)	81 (68.6)		7 (46.7)	81 (62.8)	
Duration of symptoms	≤30 days	72 (77.4)	43 (84.3)	0.324	21 (80.8)	94 (79.7)	0.899	15 (100)	100 (77.5)	0.041*†
	>30 days	21 (22.6)	8 (15.7)		5 (19.2)	24 (20.3)		0 (0)	29 (22.5)	

**Table 3:** Association of mesothelial hyperplasia and panniculitis with the demographic profile of the patients †Fisher exact test was applied.

Variables	Groups	Mesothelial hyperplasia			Panniculitis		
		Yes	No	p-value	Yes	No	p-value
		n (%)	n (%)		n (%)	n (%)	
Gender	Male	1 (14.3)	53 (38.7)	0.256†	3 (60)	51 (36.7)	0.364
	Female	6 (85.7)	84 (61.3)		2 (40)	88 (63.3)	
Age group	≤45 years	2 (28.6)	54 (39.4)	0.706	3 (60)	53 (38.1)	0.377
	>45 years	5 (71.4)	83 (60.6)		2 (40)	86 (61.9)	
Duration of symptoms	≤30 days	5 (71.4)	110 (80.3)	0.629	4 (80)	111 (79.9)	1.000
	>30 days	2 (28.6)	27 (19.7)		1 (20)	28 (20.1)	

## Discussion

When performed by a skilled operator, US-guided biopsies are a quicker method than CT-guided biopsies and pose no radiation risk to the patient or operator. Additionally, the operator can position the transducer on any axis to avoid vessels, bowels, or organs because of the multiplanar capabilities of the US [12]. The targeted greater omentum stays constant during the biopsy. The real-time visualization of the needle tip helps the process go smoothly [12,13].

Omental thickening often signifies underlying abdominal conditions such as malignancy or granulomatous inflammation [14]. Hematogenous spread from the lungs, lymphatic spread, or direct spread all have an impact on the omentum in tuberculosis. Despite the fact that surgical biopsy is the gold standard, US sonography-guided biopsy is becoming more and more popular due to its cost-effectiveness and time savings. In addition to allowing real-time needle vision during the procedure, US sonography procedures do not present with radiation risks like CT-guided biopsies [13,14].

There isn't much research on using US guidance for omental biopsies. Nine of the 11 patients in Sistrom et al.'s series, who were observed for two years, had positive results [15]. Using a 20G or 22G spinal needle for tiny needle aspirates, an 18G core biopsy needle for biopsy, or both, Gottlieb et al. obtained a sensitivity of 93% and a specificity of 100% in 54 extra-visceral masses in 52 patients. Four percent of the patients had non-diagnostic samples, and there were no problems from the surgery [16].

A malignant disease's peritoneal seeding typically denotes an advanced stage and a poor prognosis. Another significant and common condition affecting the peritoneum is tuberculous peritonitis. It may occur in less than 4% of patients suffering from pulmonary tuberculosis [16,17].

In clinical practice, a timely and accurate diagnosis of peritoneal illness is essential [18]. The particular histological diagnosis in patients with metastatic cancer determines the choice of chemotherapeutic drugs. Additionally, one of the few diffuse peritoneal disorders for which there are successful treatment options is tuberculous peritonitis. It can occasionally be difficult to distinguish between peritoneal carcinomatosis and persistent inflammation, especially in tuberculous peritonitis, based purely on imaging data [19]. Peritoneal biopsy might be the sole diagnostic choice in situations where the primary is unclear and the CT scan merely shows peritoneal thickening [20]. A few techniques, including open surgery, laparoscopy, and image-guided biopsy, are used to retrieve peritoneal tissues. These days, the gold standard diagnostic for the pathologic diagnosis of peritoneal illness is a laparoscopic peritoneal biopsy. Compared to open biopsy, laparoscopic peritoneal biopsy has a comparatively good diagnostic yield and is less invasive [21,22].

However, the use of mechanical and thermal instruments, as well as the induction of pneumoperitoneum and trocar insertion, pose a risk to the patients during this treatment. Thus, peritoneal biopsy guided by US may be a desirable substitute technique. The diagnostic utility of US-guided peritoneal biopsy has been the subject of numerous investigations [9]. 

## Conclusions

The results of this study demonstrate that US-guided omental biopsy is an invaluable diagnostic intervention for patients presenting with omental thickening. By utilizing real-time imaging, high diagnostic accuracy can be achieved while maintaining a superior safety profile that minimizes the risk of bowel perforation or significant hemorrhage. This study confirms that US-guided biopsy serves as a reliable bridge between clinical suspicion and definitive histopathological diagnosis, particularly in cases of suspected peritoneal carcinomatosis or tuberculosis.

In conclusion, the high yield and cost-effectiveness of this procedure make it an ideal first-line approach. Its ability to provide a tissue diagnosis with minimal patient discomfort and downtime warrants its prioritization over more invasive surgical options. We believe these findings will encourage the wider adoption of US-guided techniques in routine clinical practice for better patient outcomes.
